# Stimulation of cell invasion by the Golgi Ion Channel GAAP/TMBIM4 via an H_2_O_2_-Dependent Mechanism

**DOI:** 10.1016/j.redox.2019.101361

**Published:** 2019-10-22

**Authors:** Nuno Almeida, Guia Carrara, Carlos M. Palmeira, Ana S. Fernandes, Maddy Parsons, Geoffrey L. Smith, Nuno Saraiva

**Affiliations:** a CBIOS, Universidade Lusófona Research Center for Biosciences & Health Technologies, Campo Grande 376, Lisbon, 1749-024, Portugal; b Department of Pathology, University of Cambridge, Cambridge, CB2 1QP, UK; c Department of Life Sciences, University of Coimbra, Center for Neurosciences and Cell Biology, University of Coimbra, Coimbra, Portugal; d Randall Centre for Cell and Molecular Biophysics, King's College London, Guys Campus, London, SE1 1UL, UK

**Keywords:** Calcium, Cell invasion, TMBIM, Golgi apparatus, Hydrogen peroxide, Metabolism, hGAAP, Human Golgi Anti-Apoptotic Protein, SOCE, Store-Operated Ca^2+^ Entry, TMBIM, transmembrane Bax (Bcl-2-associated X protein) inhibitor-1 motif-containing protein

## Abstract

The mechanisms by which the Golgi apparatus (GA) impacts on cell invasion are poorly understood. The human Golgi Anti-Apoptotic Protein (hGAAP, also known as TMBIM4) is a highly conserved Golgi cation channel that modulates intracellular Ca^2+^ fluxes. Human GAAP is expressed in all human tissues, is essential for cell viability and provides resistance against a range of apoptotic stresses. Furthermore, hGAAP enhances adhesion and cell migration by increasing the turnover of focal adhesions due to activation of store-operated Ca^2+^ entry.

Here, we describe a GA-derived mechanism that controls cell invasion. The overexpression of hGAAP stimulates 3-dimensional proteolytic cell invasion by a mechanism that is dependent on the accumulation of intracellular hydrogen peroxide_,_ which might be produced by the hGAAP-dependent stimulation of mitochondrial respiration.

These findings provide new insight into the complex mechanisms by which Ca^2+^ and reactive oxygen species signaling contribute to cell invasion and to the role of the GA in these processes.

## Highlights

• The conserved Golgi Ion channel GAAP/TMBIM4 promotes proteolytic cell invasion.
• GAAP/TMBIM4 overexpression enhances cell adhesion and colonization *in vivo*.
• GAAP/TMBIM4 promotes mitochondrial respiration and H_2_O_2_ accumulation.
• Cell invasion stimulation by GAAP/TMBIM4 is H_2_O_2_ dependent.

## Graphical abstract

**Figure:**
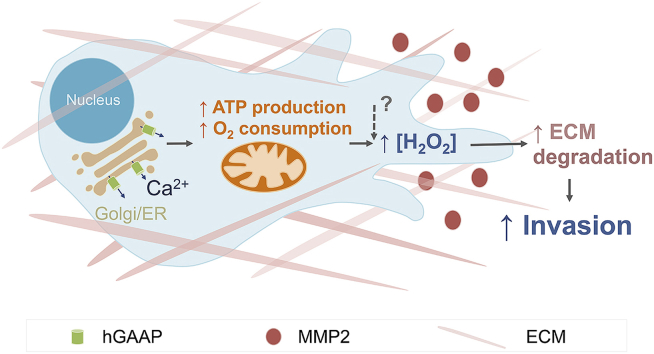

## 1 Introduction

Cell motility, and specifically cell invasion, plays an important role in physiological phenomena such as immune cell infiltration and embryogenesis, but also in pathological processes like tumor growth and metastasis. In cancer, cell invasion is generally an important process for the dissemination of cells from the primary tumor site to distant tissues [[1], [2], [3]]. While the severity and mortality of most cancers are frequently related to their ability to spread and invade other parts of the organism, the pharmacological approaches for oncological diseases rely mainly in anti-proliferative or cytotoxic drugs [4]. Only few and poorly effective strategies are available to control cancer spread and progression. This is partially due to the high complexity of the systems that control cell invasion and the variety of mechanisms used [1].

Cell invasion involves intricate coordination of adhesion and cytoskeletal remodeling that can be influenced by various cellular processes including metabolism, intracellular and extracellular pH, reactive oxygen species (ROS) accumulation, Ca^2+^ fluxes and membrane trafficking [1,[5], [6], [7], [8], [9], [10]]. The complexity of these regulatory mechanisms is increased by the interplay between several of the abovementioned elements. As an example, Ca^2+^ signaling can regulate key cellular events that are fundamental to an invading cell, such as mitochondrial metabolism, cytoskeleton remodeling, focal adhesion (FA) dynamics or matrix metalloproteinases (MMP) activation/secretion [[11], [12], [13]]. Additionally, several of these events can feedback into the system's complexity by inducing alterations in Ca^2+^ fluxes or ROS accumulation [14]. The interplay between ROS (specially H_2_O_2_) and Ca^2+^ is particularly significant in this context. Both can act as second messengers and can impact on each other [[15], [16], [17]]. Calcium entering the mitochondrion from the ER or the cytoplasm can alter O_2_ consumption and ATP production, which in turn can directly alter the levels of intracellular ROS [[17], [18], [19], [20]]. Despite their importance, the mechanisms by which Ca^2+^ and ROS contribute to cell invasion are not fully understood.

Emerging evidence suggests that the Golgi apparatus (GA) can directly or indirectly affect intracellular Ca^2+^ fluxes, ROS, metabolism, intra-organelle pH, protein and membrane trafficking and thereby contribute to cell migration/invasion [[21], [22], [23], [24], [25]]. Although these roles have been ascribed to the GA, the intricate molecular mechanisms involved in the control of such processes are still mostly unidentified.

The human Golgi Anti-Apoptotic Protein (hGAAP), also known as transmembrane Bax (Bcl-2-associated X protein) inhibitor-1 motif-containing protein 4 (TMBIM4) is a highly conserved GA cation-selective ion channel that controls the Ca^2+^ filling state of the GA and ER and regulates store-operated Ca^2+^ entry (SOCE) [26,27]. The ubiquitous expression of GAAP across all human tissues [[28], [29], [30]], suggests that its role might be fundamental to the function of a wide variety of cell types. Indeed, hGAAP was proposed to be a housekeeping protein [28,31]. Members of the TMBIM family of ion channels are located in various organelles, and are structurally different from all other ion channels described to date [32,33]. The mechanisms controlling ion flow in TMBIM proteins are not fully understood, but recent structural data obtained from a bacterial TMBIM suggests that intracellular pH might participate in the opening of the pore [34]. Spontaneous channel activity of single purified GAAP of virus origin was recorded by electrophysiological experiments, consistent with a passive leak mechanism [26]. Human GAAP overexpression was shown to enhance SOCE, leading to activation of the protease calpain 2 near the plasma membrane, resulting in greater FA turnover and increased migration [27].

Here, we provide evidence that hGAAP also enhances mitochondrial metabolism and proteolytic cell invasion in an H_2_O_2_-dependent manner. This study provides new insight into mechanisms by which Golgi-associated proteins act to control Ca^2+^ and ROS production to facilitate invasive signaling.

## 2 Material and Methods

### 2.1 Cell culture and transfection

Human osteosarcoma U2-OS and breast cancer MCF7 cell lines were grown in Dulbecco's Modified Eagle Medium (DMEM - BioWest). Medium was supplemented with 10% foetal bovine serum (FBS) (BioWest), 100 units/ml penicillin and 100 μg/ml streptomycin. Plasmid transfections were carried out using Lipofectamine LTX (Invitrogen) or TransIT LT1 (Mirus) according to the manufacturer's instructions and plasmid-transfected cells were selected using G418 (Gibco) to generate polyclonal cell lines. The siRNA oligonucleotide duplexes (Invitrogen) were transfected using Oligofectamine (Invitrogen) according to the manufacturer's instructions. Sequences of the hGAAP-specific siRNA1 and 2 were described [28]. A siRNA for GFP was used as control [27]. Cells were used 36–40 h after transfection.

### 2.2 Plasmids and stable cell lines

Polyclonal U2-OS cells expressing the control plasmid (neo), hGAAP or hGAAP Ctmut with C-terminal mutations (E233AVNKK238 to A233AVAAA238), each with a C-terminal HA tag, were described [28,35]. Immunoblot, reverse transcription-quantitative polymerase chain reaction (RT-qPCR) and immunofluorescence were used to control for gene expression and subcellular localization. The HyPerRed and HyPerRed-C199S [36] were obtained from Addgene.

### 2.3 Immunoblotting

Cells were lysed on ice in lysis buffer (50 mM Tris-HCl pH 7.5, 100 mM NaCl, 2 mM EDTA, 1% CHAPS, protease and phosphatase inhibitor cocktails (Roche)). The lysates were cleared by centrifugation (15,000×*g*, 15 min), resolved using NuPAGE® Novex 4–12% Bis-Tris gels (Invitrogen) and transferred onto a nitrocellulose membrane. Antigen-antibody complexes were detected using horseradish peroxidase (HRP)-conjugated secondary antibodies (Sigma-Aldrich). The primary antibodies used were: anti-HA (Sigma, 1:10,000), anti-α tubulin (Millipore, 1:10,000).

### 2.4 RT-qPCR

The levels of endogenous hGAAP mRNA in U2-OS cells following siRNA transfection were measured by RT-qPCR using a ViiA 7 Real-Time PCR System (Life Technologies), fast SYBR Green Master Mix (Applied Biosystems), hGAAP primers (Fwd: AGGACGACTTCAACTATGGC, Rev: CCAGAAACCGTAACAGGTGC) and Glyceraldehyde-3-Phosphate Dehydrogenase (GAPDH) primers (Fwd: ACCCAGAAGACTGTGGATGG, Rev: TTCTAGACGGCAGGTCAGGT). Total cellular RNA was extracted from transfected cells 24 h and 48 h post siRNA transfection, using a RNeasy mini kit (QIAGEN) and 1 μg of total RNA was reverse transcribed using Superscript III reverse transcriptase according to the manufacturer's protocol (Invitrogen). For relative quantification analysis, amplified hGAAP was normalized to endogenous GAPDH amplified from the same sample in triplicates. Experiments were performed in biological triplicates and conducted three times.

### 2.5 In vitro cell invasion and migration assays

The chemotactic invasion and migration was evaluated in 24-well plates with transwell inserts with transparent PET membranes containing 8-μm pores (BD Falcon) using 10% FBS as chemoattractant. The invasion assay was performed as described for chemotactic migration measurements, but in this case the membrane filter was overlaid with Matrigel™ (BD Biosciences) diluted in serum-free medium (1:30 dilution) [37,38]. Non-migrating cells were removed from the upper side of the inserts with a cotton swab. Cells that migrated to the underside of the inserts were fixed with 96% ethanol and stained with 0.1% crystal violet. The number of cells per field was counted for at least 10 fields on an Olympus BX51 inverted microscope using a x20 or x40 objective.

### 2.6 In vivo cell invasion assay

This study was performed under the Research Project “The role of the human Golgi-anti apoptotic protein in cell invasion”, which was appraised by the Animal Welfare Body of the Champalimaud Foundation and licensed by the Portuguese competent authority, Direcção Geral de Alimentação e Veterinária (DGAV) under Ref. 0421/000/000/2017. Housing, husbandry and procedures were designed and performed according to Directive 63/2010/EC and the 3Rs Principle.

**Animals**: For this study, 8-week old NOD. CB17-Prkdcscid/J (known as NOD scid) male mice were used. Mice were purchased from The Jackson Laboratory, Bar Harbour, ME, USA and were bred in the Champalimaud Foundation Vivarium. This study was conducted in the Specific and Opportunistic Pathogen-Free (SOPF) Suite according to FELASA recommendations Mice are housed in individually ventilated cages (Tecniplast Green Line IVC Sealsafe PLUS Mouse) and handled inside ventilated stations (Tecniplast Aria). All cages are garnished with corncob bedding, red plastic igloos for shelter, and crinkled paper for nesting. Mice were fed an autoclaved standard pelleted rodent diet (RM3, Special Diet Services) and drank autoclaved tap water, both supplied *ad libitum*.

**Cell staining and detection**: U2-OS cells overexpressing hGAAP (hGAAP) and control cells (neo) were stained with CellTrace™ CFSE (Thermo Fisher) and Vybrant™ DiD (Thermo Fisher), respectively, according to the manufacturer instructions.

A short pilot experiment was performed on 5 mice to evaluate the number of stained cells in the lung at 5 different time points post injection. From the data obtained, 8 h post-injection was selected as the best time-point for lung harvest. After this, five mice were used per experiment and two independent experiments were performed. Each animal was injected with 1 × 10^6^ cells of each cell line (2 × 10^6^ in total) in the tail vein under isoflurane anesthesia. Eight h post injection, the animals were sacrificed by cervical dislocation and the lungs were harvested. A single cell suspension of the entire lung tissue was obtained using a 70 μm cell strainer. The erythrocytes present in the cell suspension were lysed using two incubations with Erythrocyte Lysis Buffer (155 mM NH_4_Cl, 12 mM NaHCO_3_ and 0.1 mM EDTA, pH 7.3). Cells were analyzed by flow cytometry using a FACSFlow (BD Biosciences) to detect CFSE and Vybrant DiD stained cells. A total of 2 × 10^6^ cells were analyzed for each sample. Data analysis was performed using FlowJo.

### 2.7 Fluorescent gelatin degradation assay

Fluorescent gelatin-coated cover slips were prepared as described [39]. Briefly, coverslips were coated with thin layers of Oregon Green 488-conjugated gelatin (Molecular probes), cross-linked with 0.5% glutaraldehyde, incubated with 5 mg/ml NaBH_4_, washed with PBS and incubated in 70% ethanol. Cells were seeded on gelatin-coated coverslips at a density of 4 × 10^4^ cells per well in a 24 well plate, in complete medium. After 20 h cells were fixed with 4% paraformaldehyde. Image acquisition was performed on a wide-field Zeiss Axiobserver microscope with a x40 objective and ZEN software. The gelatin degradation percentage (per image) was measured using Image J software and was then normalized to the number of cells to obtain a normalized degradation value.

### 2.8 Gelatin zymography

Protease activity of secreted MMP2 and MMP9 in the culture medium of U2-OS cells was analyzed using a gelatin zymography assay as described [40]. Cells (3 × 10^5^ cells) were seeded on MatrigelTM–coated 6-well plate and allowed to grow for 24 h. Cells were incubated for 24 h with FBS-free medium. The conditioned medium was collected, cleared by centrifugation, concentrated using Vivaspin concentrators (30 kDa cut-off, Sartorius Stedim Biotech) and resolved in a 10% SDS–polyacrylamide gel copolymerised with 0.1% (w/v) gelatin. The gel was incubated in renaturing buffer (25% v/v Triton X-100 in water) for 30 min at room temperature and developing buffer (50 mM Tris–HCl, pH 7.8, 0.2 M NaCl, 5 mM CaCl_2_, and 0.02% Brij 35) at 37 °C for 16 h. Inclusion of EDTA in the developing buffer inhibits MMP activity allowed the confirmation of the bands specificity. The gel was stained with Coomassie Blue R-250 and imaged with a ChemiDoc XRS (Bio-Rad, USA). The semi-quantitative densitometry of MMP-2 and MMP-9 bands was performed using ImageJ as described [40]. Total protein content of concentrated supernatant and cell lysate was measured by Bradford assay (BioRad) prior to gel loading to avoid variations in cell number/mass, total level of protein secretion or loss during concentration.

### 2.9 ROS and H_2_O_2_ measurements

The overall levels of intracellular ROS were evaluated using CellROX (Molecular Probes), a cytoplasmic cell-permeant dye that becomes fluorescent upon oxidation by several ROS. Briefly, 1.5 × 10^5^ cells/well of each U2-OS cell line were seeded in T25 flasks. Cells were washed with PBS, detached using Accutase (Gibco), washed with pre-conditioned medium and stained with 5 μM CellROX for 30 min at 37 °C according to the manufacturer's instructions. Propidium Iodide (PI) was used as a live/dead stain. Tert-Butyl hydroperoxide (TBHP) and N-acetylcysteine (NAC) (Sigma) were used as controls. The far-red intensity (excitation/emission: 644/665 nm) of each cell line population was analyzed by flow cytometry using a Cytek FACScan DxP8 and analyzed using the MoFlo software, following doublet cell discrimination and PI stain dead cell exclusion.

The genetically encoded sensor HyPerRed was used to measure intracellular levels of H_2_O_2_ as described [36]. U2-OS cell lines were seeded (3 × 10^5^ cells/well) on 35 mm glass-bottom dishes and left to settle for 24 h. Cells were then transfected with 1 μg of the HyPerRed plasmid for 12 h using TransIT LT1 according to manufacture's instructions. In preparation for live cell imaging, cells were incubated for 2 h with Hank's Balanced Salt Solution (HBSS) supplemented with 20 mM HEPES - NaOH pH 7.4 at 37 °C. Image acquisition was performed within an environmental chamber at 37 °C using a wide-field microscope (LSM 5 PASCAL, Zeiss), with a x10 objective and a camera (AxioCam HRm; Carl Zeiss) with Ex: 570 nm/Em 620 nm. To control for possible interference due to variations in intracellular pH, the same protocol was applied with a H_2_O_2_ insensitive but pH sensitive mutant version of the sensor (HyperRed-C199S). The mean fluorescence intensity analysis was performed and normalized for cell area using Image J (NIH).

### 2.10 Mitochondrial metabolism

The assessment of oxygen consumption rate (OCR) in U2-OS cell lines was undertaken as described [41]. Briefly, 5 × 10^4^ cells/well of each U2-OS cell line were seeded in 24-well Seahorse XF Cell Culture Plates in 500 μL of complete medium and left for 24 h at 37 °C. The Seahorse XFe24 Sensor Cartridges were filled with Seahorse XF Calibrant Solution pH 7.4 and left overnight to hydrate in a 37 °C incubator without CO_2_. Just prior to the experiment all the growth medium was removed and the cells were washed with fresh Seahorse Assay medium (Seahorse XF DMEM Medium, pH 7.4 supplemented with 10 mM glucose, 1 mM pyruvate and 2 mM l-glutamine) pre-warmed at 37 °C. A final volume of 500 μL Seahorse Assay medium was left in the wells for the experiment. To equilibrate temperature and pH the plate was incubated in a 37 °C incubator lacking CO_2_ for 1 h prior to the assay. To generate OCR, oligomycin (1 μM), FCCP (1 μM) and antimycin A (0.1 μM) were injected in the wells by the Seahorse XFe24 Analyzer and 3 measurements of OCR were taken in each cycle of the analysis. At the end of the assay the medium was removed and the cells were washed with cold PBS and lysed with RIPA buffer for protein quantification. Protein was quantified using a BCA assay. The final values were calculated and expressed with normalization of protein content using Agilent software Wave.

### 2.11 Statistical analysis

Statistical analyses were performed by two-tailed Student's t-test, Paired *t*-test, or One-way ANOVA with Tukey's test using the GraphPad Prism statistical analysis software. p < 0.05 was considered statistically significant.

## 3 Results

### 3.1 hGAAP induces cell invasion and matrix degradation

**Fig. 1:**
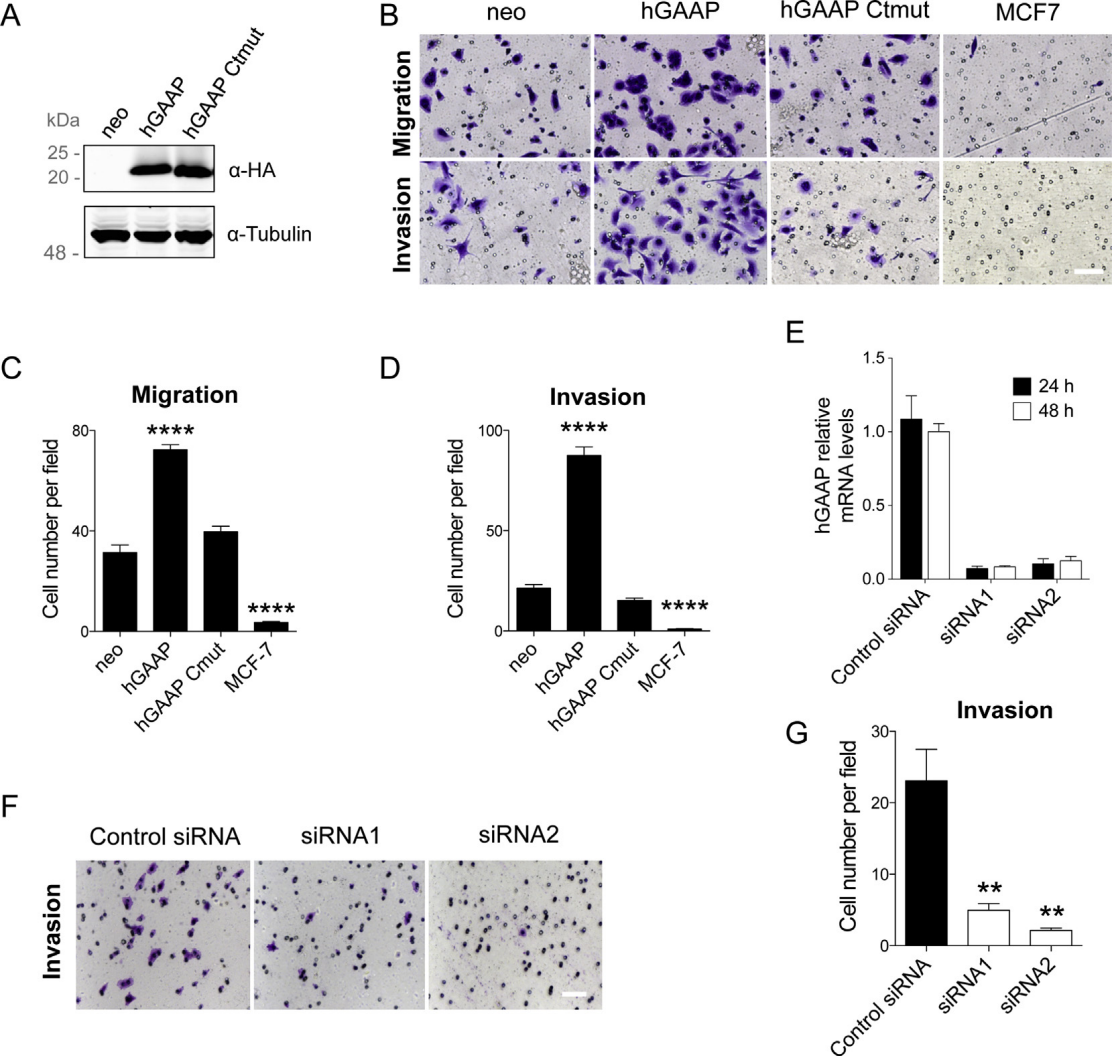
**hGAAP overexpression or knock down affects *in vitro* cell invasion.** The migration and invasion of cells overexpressing hGAAP, a C-terminal mutant of hGAAP, or empty vector (neo) and of cells knocked down for hGAAP was quantified by transwell assays after 8 h. Cell invasion conditions were created by the addition of a layer of matrigel to the transwell membrane. (A) Immunoblot of U2-OS cells with anti-HA Ab shows expression of HA-tagged hGAAP and hGAAP Ctmut in stable cell lines, but not in cells expressing control plasmid (neo). (B, F) Representative images of migrating/invading cells. (C, D and G) Summary results (shown as mean ± SD from a representative experiment from 3 independent experiments) show the number of invading or migrating cells, **p < 0.01 and ****p < 0.0001 (Student's *t*-test compared to neo control cells or control siRNA). (E) endogenous hGAAP mRNA expression levels were determined 24 and 48 h after siRNA transfection by RT-PCR using hGAAP specific primers.

Previously, hGAAP expression was shown to increase random cell migration on 2D surfaces by activating SOCE and calpain2-dependent FA turnover [32]. To determine whether hGAAP overexpression also played a role in directed migration, U2-OS cells expressing empty vector (neo), hGAAP or mutant of hGAAP in which a series of charged residues at the C terminus that are essential for regulating cell adhesion, apoptosis and Ca^2+^ homeostasis [3,15,[17], [18], [19]] are mutated to uncharged amino acids (hGAAP Ctmut) were used (Fig. 1A). Non-invasive MCF7 cells were included as a control. The results showed a significant increase in directed migration through uncoated (migration) and 3D Matrigel coated (invasion) chambers in cells overexpressing hGAAP, but not those expressing hGAAP Ctmut (Fig. 1B–D). Moreover, U2-OS cells silenced for endogenous hGAAP (Fig. 1E) exhibited over 3-fold lower invasion than control cells, further confirming the requirement for hGAAP in this process (Fig. 1F and G).

**Fig. 2:**
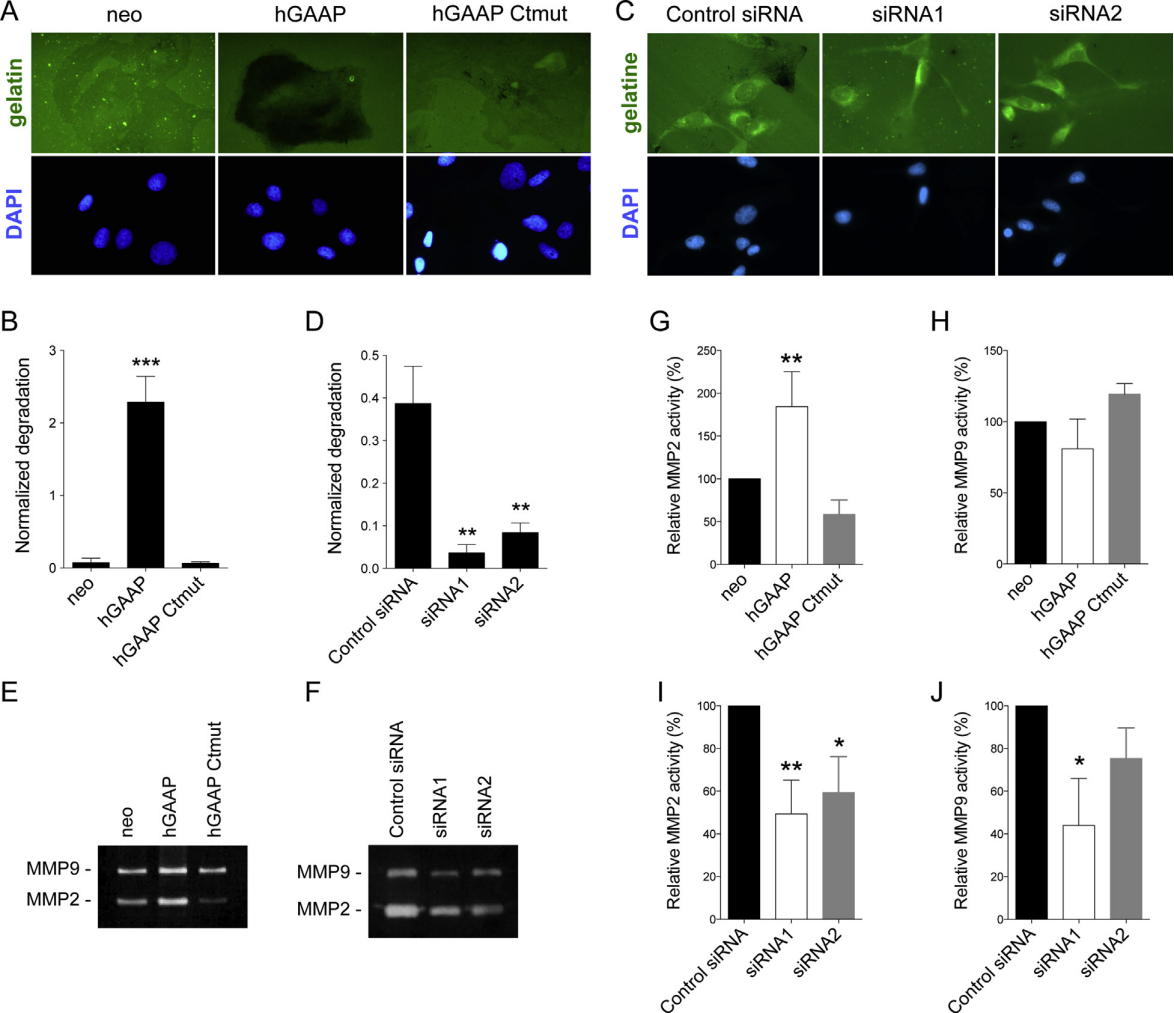
**hGAAP overexpression induces extracellular proteolytic degradation and MMP2 activity.** (A, B) Cells overexpressing hGAAP, a C-terminal mutant of hGAAP or empty vector (neo), and cells transfected with hGAAP specific or control siRNAs (C, D) were seeded on fluorescent gelatin-coated cover slips to detect extracellular proteolytic activity. (B–D) Summary results (shown as mean ± SD from 3 independent experiments) show normalized gelatin degradation, ***p < 0.001 (Student's *t*-test compared to neo). (E–J) MMP2 and MMP9 activity from the supernatants of cells overexpressing hGAAP (E) or cells where hGAAP was knocked down by siRNA (F) was measured using a gelatin zymography assay. (G–J) Summary results (means ± SD from 4 to 5 independent experiments) show relative MMP activity, *p < 0.05, **p < 0.01 (Student's *t*-test compared to neo).

Cells can invade the extracellular matrix (ECM) using different strategies that may vary according to pore size, matrix elasticity, cellular contractility and adhesion [1,42]. To determine if the mechanism underlying hGAAP-induced cell invasion involved increased ECM proteolytic degradation, cells were analyzed using a fluorescent gelatin degradation assay. Quantification of images revealed that cells overexpressing hGAAP showed significantly higher degradation of matrix when compared to hGAAP Ctmut overexpression or control neo cells (Fig. 2A and B). Similar results were obtained when hGAAP was overexpressed in the non-invasive breast cancer cell line MCF-7 (Fig. S1). Conversely, cells silenced for hGAAP using siRNAs showed a significant reduction in matrix degradation (Fig. 2C and D). Activity levels of two extracellular proteases (MMP2 and MMP9) responsible for proteolytic activity during cell invasion were assessed by gelatin zymography. Levels of active MMP2 were increased upon hGAAP overexpression, and, conversely, were reduced upon hGAAP knock down, whereas no pronounced differences were observed on the amount of active MMP9 (Fig. 2E–J). Taken together, these data demonstrate that hGAAP promotes proteolytic cell invasion potentially through increased levels of active MMP2.

### 3.2 hGAAP induces cell adhesion and invasion to the lung

**Fig. 3:**
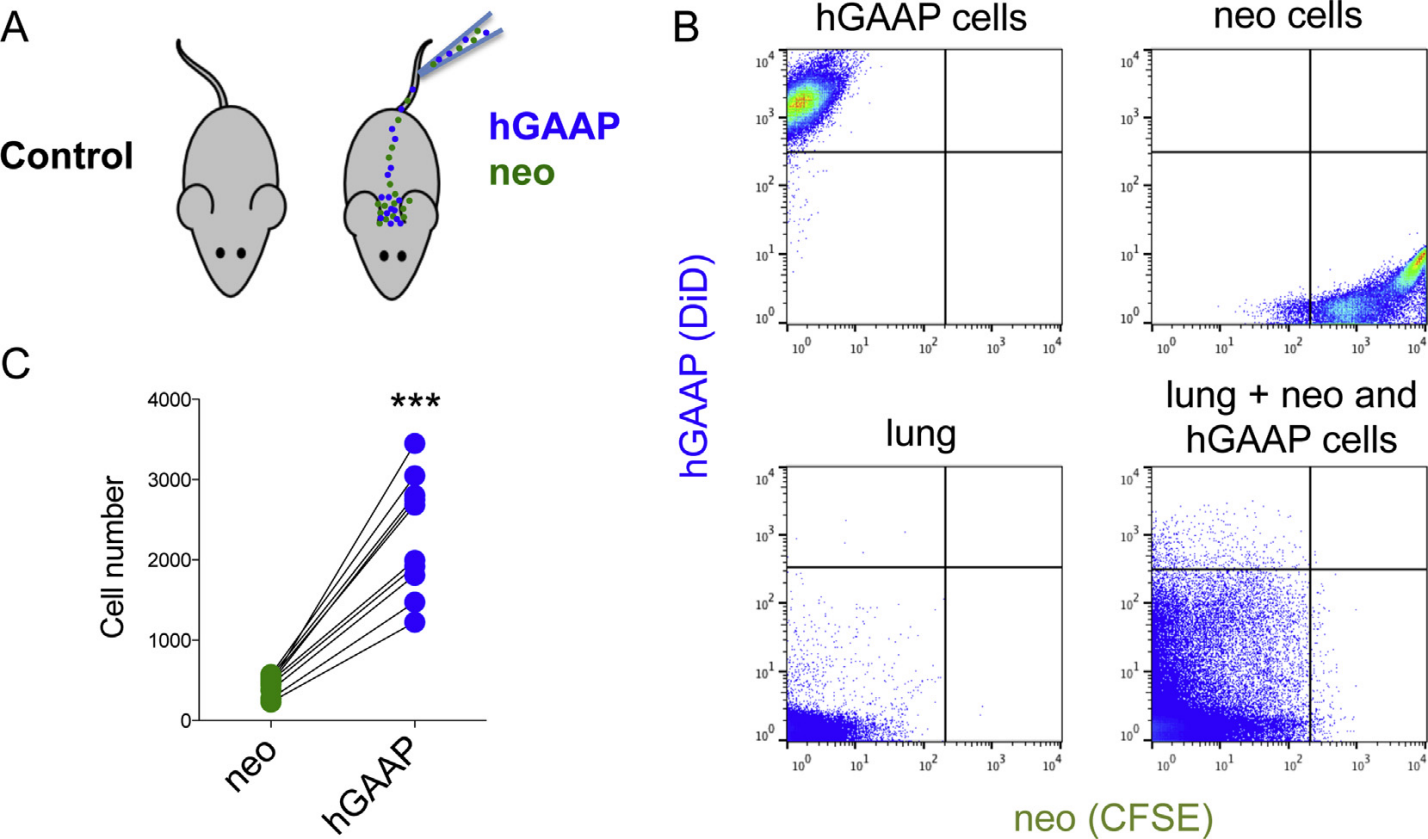
**hGAAP overexpression increases *in vivo* cell adhesion and invasion into the lung tissue.** (A) The *in vivo* lung adhesiveness and invasiveness of U2-OS cells overexpressing hGAAP or control plasmid was assessed by co-injecting pre-stained cells (hGAAP cells stained with DiD and neo cells stained with CFSE) into the tail vein of NOD SCID mice. (B) The lungs were collected 8 h post cell injection and analyzed by flow cytometry to detect the injected cells. (C) Summary results (10 animals from 2 independent experiments) show the number of fluorescent cells recovered from the lungs, lines connect the values that were obtained in each animal, ***p < 0.001 (Paired *t*-test, compared to neo).

To address the significance of these findings in an *in vivo* context, cells overexpressing hGAAP or control cells with endogenous levels of hGAAP (neo) were stained with different fluorescent dyes and co-injected into the tail vein of immunedeficient NOD scid mice (Fig. 3A). Lungs were collected 8 h post injection and the number of fluorescent cells present in the lung was determined by flow cytometry (Fig. 3B). Data demonstrated significantly higher numbers of hGAAP overexpressing cells were resident within the lung tissue compared with the neo control cells (Fig. 3C). Taken together, these data support a role for hGAAP overexpression in enhancing cell adhesion and colonization *in vivo*.

### 3.3 hGAAP overexpression promotes mitochondrial respiration and increases intracellular H_2_O_2_

**Fig. 4:**
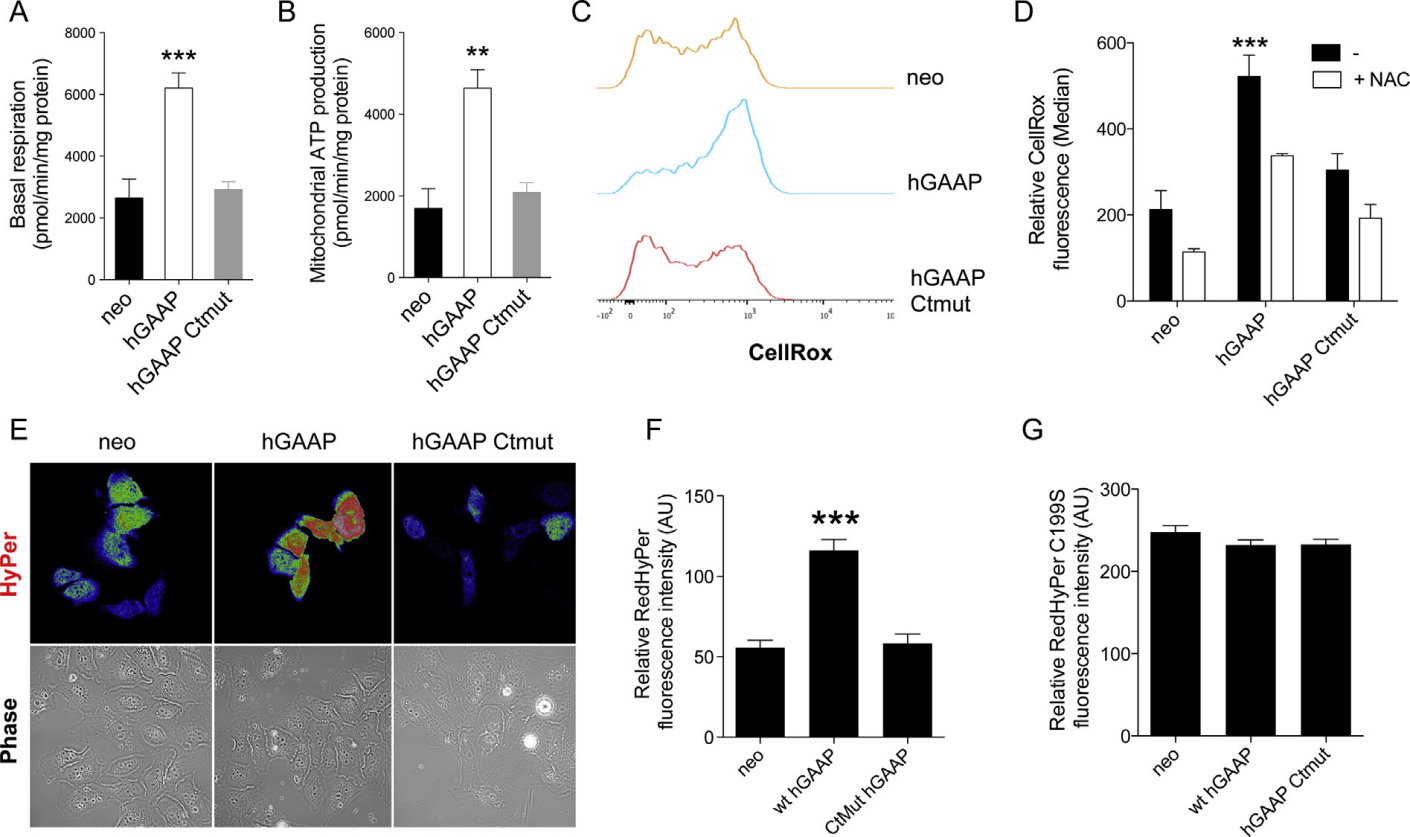
**hGAAP overexpression increases mitochondrial respiration and intracellular levels of ROS and specifically of H**_**2**_**O**_**2**_**.** The impact of hGAAP overexpression in mitochondrial metabolism of cells overexpressing hGAAP, a C-terminal mutant of hGAAP or empty vector (neo) was analyzed using the seahorse XF mito stress test (A–B). (A) Basal respiration and (B) mitochondrial ATP production were calculated. Summary results show means ± SD from a representative experiment from 3 independent experiments. (C) The intracellular levels of ROS were determined using the fluorescent broad ROS sensor CellRox by flow cytometry. (D) Summary results (shown as mean ± SD from a representative experiment out of 3 independent experiments) show median fluorescence intensity. (E–F) Relative H_2_O_2_ levels were determined using the H_2_O_2_ sensor HyPerRed. (G) A mutated version of the HyPerRed sensor (HyPerRed C199S) that renders the protein insensitive to H_2_O_2_ but preserves the sensitivity to pH changes was used to control for a possible effect of intracellular pH changes in HyPerRed fluorescence. (F–G) Summary results (shown as mean ± SD from cells from 3 independent experiments) show median HyPerRed fluorescence intensity. **p < 0.01 and ***p < 0.001 (Student's *t*-test compared to neo).

An increased ER and GA Ca^2+^ flow into the mitochondrion can alter mitochondrial metabolism, typically leading to higher ATP production and O_2_ consumption [[18], [19], [20],^43^]. Previous reports have also demonstrated that a close physical link between the ER/GA and mitochondria exist and that Ca^2+^ is able to flow from the ER/GA to mitochondria, thus affecting mitochondrial respiration and ROS production [19,44,45]. We postulated that GAAP channel activity at the GA enhanced by over-expression allows draining of Ca^2+^ from the GA/ER thereby reducing the Ca^2+^ content of GA/ER stores [46], could lead to an increase in mitochondrial metabolism. The potential impact of hGAAP overexpression on mitochondrial metabolism was analyzed using a Seahorse XF Cell Mito stress test. Data revealed a robust increase in ATP production and O_2_ consumption when hGAAP was overexpressed (Fig. 4A–B), suggesting that hGAAP expression promotes an increase in mitochondrial respiration.

Increases in mitochondrial O_2_ consumption and ATP production frequently lead to an increase in the production of ROS [44]. ROS play an important role in cell migration and in particular cell invasion-related processes [47,48]. To determine whether the observed GAAP overexpression correlated with an increase in ROS levels, cells were assessed for ROS using CellROX. Data demonstrated that ROS levels were significantly higher in cells overexpressing hGAAP compared with neo control cells (Fig. 4C–D). Conversely, the overexpression of the hGAAP C-terminal mutant did not elicit an increase in intracellular ROS (Fig. 4C–D). The free radical scavenger, NAC, included as a control, effectively inhibited hGAAP-mediated increase in global ROS levels (Fig. 4D).

Hydrogen peroxide plays a role controlling several cell motility-related processes and is a key regulator of cancer cell migration [[48], [49], [50], [51], [52], [53], [54]]. Considering the accumulated evidence for a role of H_2_O_2_ in cell invasion, the impact of hGAAP overexpression in H_2_O_2_ accumulation was assessed. The genetically encoded sensor HyPerRed was used to measure intracellular levels of H_2_O_2_ in live cells. Resulting data revealed that hGAAP overexpression significantly and specifically increased H_2_O_2_ intracellular levels (Fig. 4E–F). Analysis of parallel samples using a mutated version of the HyPerRed sensor that renders the protein insensitive to H_2_O_2_ but preserves the sensitivity to pH variations, showed no change between cell lines (Fig. 4G) indicating a specific GAAP-dependent increase in H_2_O_2_ levels.

### 3.4 hGAAP-induced cell invasion and ECM degradation is H_2_O_2_ dependent

**Fig. 5:**
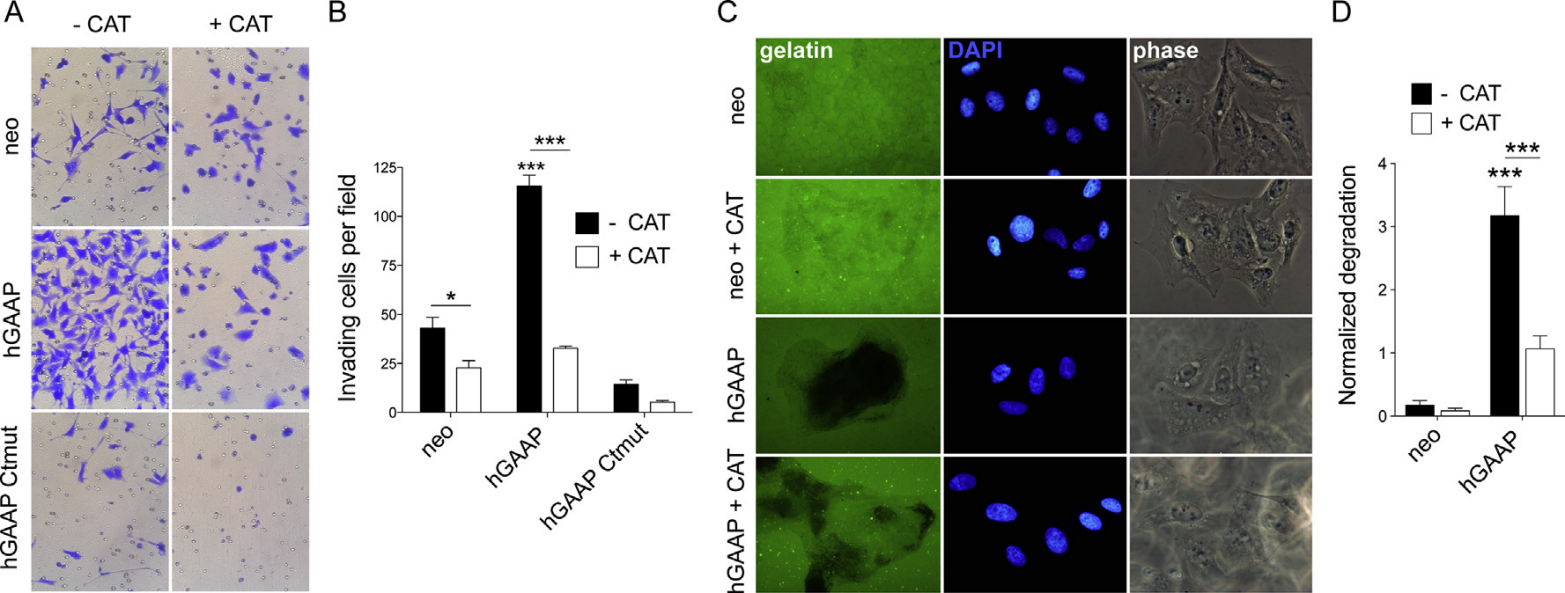
**hGAAP-induced cell invasion and extracellular proteolytic degradation is reverted by catalase.** Cell invasion (A–B) and extracellular proteolytic degradation (C–D) in the presence and absence of catalase (CAT) were evaluated in U2-OS cell lines using (A–B) matrigel-coated transwell invasion assay and (C–D) fluorescent gelatin degradation assays, respectively. (B and D) Summary results (shown as mean ± SD from cells from 3 independent experiments) show (B) the average number of invading cells and (D) normalized gelatin degradation, *p < 0.05, ***p < 0.001 (One-way ANOVA, Tukey's Test).

To determine whether the observed hGAAP-induced H_2_O_2_ accumulation played a role in hGAAP-induced cell invasion and extracellular proteolytic activity, cells were incubated with catalase to lower the overall concentration of H_2_O_2_ in the cell before being subjected to cell invasion and fluorescence gelatin degradation assays. The addition of catalase restored the hGAAP-induced cell invasion phenotype to levels seen in control cells (Fig. 5A–B) and partially reverted the increase in extracellular proteolytic activity observed when hGAAP was overexpressed (Fig. 5C–D). These data suggest that H_2_O_2_ is responsible for the observed hGAAP-induced cell invasion and extracellular proteolytic phenotypes.

## 4 Discussion

The GA-resident cation channel hGAAP is part of a bigger family (TMBIM) of highly conserved membrane proteins. The roles played by members of this family in critical aspects of cellular biology (Ca^2+^ fluxes, adhesion/migration, apoptosis, unfolded protein response - UPR, among others) [27,[55], [56], [57], [58]] together with the high level of conservation and house keeping roles of some members, highlight the importance of this group of poorly understood proteins.

Here we describe a novel mechanism controlling cell invasion by a protein resident in the GA. Changes in the expression levels of hGAAP induce a marked effect on 3D proteolytic cell invasion. Overexpression of hGAAP leads to pronounced changes in the mitochondrial metabolic status with an increase in mitochondrial O_2_ consumption and ATP production accompanied by greater ROS accumulation (Fig. 4) [43]. All above-mentioned effects were inhibited by the mutation of critical residues near the cytosolic C terminus (Ctmut hGAAP) that abrogate hGAAP-induced Ca^2+^ fluxes and all the other known roles of this protein [35]. The use of the Ctmut hGAAP served to discard the possibility of protein overexpression-induced UPR activation, changes in membrane or protein trafficking or other more subtle mechanisms to account for the observed effects.

The impact of hGAAP on cell invasion and extracellular proteolytic degradation were inhibited by the addition of catalase, supporting a central role for H_2_O_2_ as a key signaling molecule in this process (Fig. 5). We propose that hGAAP overexpression promotes the generation of H_2_O_2_ at least partially by activating mitochondrial respiration, possibly by allowing the flow of Ca^2+^ from the GA into mitochondria. Entry of Ca^2+^ into the mitochondria has repeatedly been linked with an increase in mitochondrial ATP production and O_2_ consumption [18,19,59], and with changes in cell invasion [8,43], in agreement with the data we present here. Moreover, a close physical contact exists between mitochondria and the GA/ER, enabling a direct flow of Ca^2+^ between these organelles [45,59]. Deregulation of this flow has been linked to mitochondrial metabolic alterations and consequently to ROS accumulation and to changes in cellular events including cell invasion [7,8].

The accumulation of intracellular and extracellular ROS, and specifically H_2_O_2_ is recognized to play a part in cell motility/adhesion (reviewed in Ref. [60]). Although the involvement of ROS in cell motility is clear, the mechanisms underlying these effects are not understood in many cases. Hydrogen peroxide has an important role as a diffusible second messenger, and can act through oxidation of cysteine thiols [17]. Regarding cell motility, H_2_O_2_ can function as a chemoattractant [49,61], alter the expression or directly oxidize cell motility-related proteins [52,62], indirectly affect cytoskeleton remodeling [63], or modify MMP activation [64]. The specific mechanism of action of hGAAP-induced H_2_O_2_ on cell invasion and extracellular proteolytic activity remains to be elucidated.

Whilst various reports have recognized the involvement of the GA in regulating cell invasion, this is the first report of a GA resident ion channel controlling cell invasion and mitochondrial metabolic status. The positioning of the GA at the front of migrating cells mediates the delivery of new membrane and adhesion proteins to the leading edge and is closely linked with cell polarity and directional migration [[65], [66], [67]]. Several GA proteins have been linked with cell migration and are associated with cancer progression. The upregulation of the GA-resident proteins GOLPH2 and GOLPH3 is associated with GA orientation and have been linked with tumorigenesis and cancer progression by promoting cell migration, invasion and adhesion as well as mitochondrial biogenesis [68,69].

Data obtained in the last two decades supports a role for ion channels and transporters in the regulation of the metastatic behavior of tumor cells [10]. However, the integrated regulation of the signaling elements involved, and the corresponding clinical outcomes are not well comprehended (reviewed in Ref. [70]).

Human GAAP overexpression in tumor cell lines (U2-OS, MCF7 and HeLa) induces pro-invasive cellular events, suggesting that hGAAP might play a role in the progression from primary non-invasive cancer to a metastatic-competent state. Since hGAAP regulates several important hallmarks of cancer (resisting cell death, deregulating cellular energetics, activating invasion & metastasis) and significant dysregulation of its expression has been reported in an increasing number of malignant tissues [32], hGAAP might affect tumor progression and metastasis. Other TMBIM members have been reported to participate in the control of cell motility and to impact metastasis formation, namely TMBIM6 (also known as Bax inhibitor 1, BI-1) [71] and TMBIM2 (also known as FAS inhibitory molecule 2, FAIM2) [32,72]. TMBIM6 is a Ca^2+^ ion channel located at the ER and has been implicated in the regulation of cellular metabolic status [71]. In contrast to this report for hGAAP, Lee et al., 2010 [47,71] described a reduction in O_2_ consumption and ROS production upon TMBIM6/BI-1 overexpression, highlighting the potentially very different roles for these structurally and evolutionarily similar proteins.

## 5 Conclusions

This is the first report describing the role of a GA-resident channel in the control of cell invasion and matrix degradation. Our findings highlight the importance of the GA in the control of cell motility and metabolism.

TMBIM proteins such as GAAP/TMBIM4 regulate various key cellular events. Dissecting such regulatory mechanisms contributes to the understanding of the fundamental cell biology processes in which these ancestral proteins participate. It will also help to define the role of these molecules in various aspects of human pathologies such as cancer progression.

## Availability of data and materials

The datasets used and/or analyzed during the current study are available from the corresponding author on reasonable request.

## Funding

This work was supported by Fundação para a Ciência e a Tecnologia, through funding UID/DTP/04567/2019 to CBIOS. GC was supported by an Isaac Newton grant. NA acknowledges his research grant attributed in the scope of the project UID/DTP/04567/2016. NS was awarded STSM grants from EU-ROS (BM1203) and EuroCellNet (CA15214) COST actions. GLS is a Wellcome Trust Principal Research Fellow.

## Authors' contributions

NS, ASF, GLS, GC and MP contributed to the conception and design. NS, GC, MP, CMP and ASF contributed to the development of methodology. NS, GC, NA and MP contributed to the acquisition of data. NS, GC, MP, GLS and ASF contributed to the analysis and interpretation of data. NS, NA, GC, GLS, MP, ASF contributed to the writing, review, and/or revision of the manuscript. NS and GLS supervised the study. All authors read and approved the final manuscript.

## Declaration of competing interest

The authors declare that they have no known competing or conflicting financial interests or personal relationships that could have appeared to influence the work reported in this paper.
