# Supplementary material for: Stimulation of cell invasion by the Golgi Ion Channel GAAP/TMBIM4 via an H2O2-Dependent Mechanism
Source: Redox Biol. 2019 Oct 22;28:101361. doi: 10.1016/j.redox.2019.101361 (PMC6838802; doi:10.1016/j.redox.2019.101361)
Supplement: Multimedia component 1 [file mmc1.docx]

**Figure S1 - hGAAP overexpression induces extracellular proteolytic degradation of MCF7 cells.** (A) MCF7 cells overexpressing hGAAP or empty vector (neo) were seeded on fluorescent gelatin-coated coverslips to detect extracellular proteolytic activity. (B) Summary results (shown as mean ± SD from 3 independent experiments) show normalized gelatin degradation.
